# Self-reported health-related quality of life is an independent predictor of chemotherapy treatment benefit and toxicity in women with advanced breast cancer

**DOI:** 10.1038/sj.bjc.6605649

**Published:** 2010-04-13

**Authors:** C K Lee, M R Stockler, A S Coates, V Gebski, S J Lord, R J Simes

**Affiliations:** 1 NHMRC Clinical Trials Centre, The University of Sydney, Camperdown, NSW, Australia; 2 Sydney Cancer Centre – Royal Prince Alfred and Concord Hospitals, Sydney, NSW, Australia; 3 International Breast Cancer Study Group, Bern, Switzerland

**Keywords:** quality of life, advanced breast cancer, treatment benefits, treatment toxicity

## Abstract

**Background:**

Baseline health-related quality of life (QL) is associated with survival in advanced breast cancer. We sought to identify patients who were less likely to respond to chemotherapy and at greater risk of toxicity on the basis of their QL.

**Methods:**

We used data from three advanced breast cancer trials in which patients (*n*=378) were treated with cyclophosphamide, methotrexate and 5-fluouracil. Patients self-rated their QL using LASA scales for physical well-being (PWB), mood, pain, nausea/vomiting, appetite and overall QL. Multivariable regression models were constructed to compare overall survival (OS), objective tumour response (OTR), adverse events (AEs) and weight loss according to grouped QL scores.

**Results:**

Physical well-being, mood, appetite and overall QL were significant univariable predictors of OS. Physical well-being and appetite remained significant after adjustment for baseline biomedical factors. Poor PWB was associated with lower OTR (odds ratio (OR)=0.21, 95% confidence interval (CI) 0.09–0.51), higher risk of non-haematological AEs (OR=3.26, 95% CI 1.49–7.15) and greater risk of weight loss (OR 2.37, 95% CI 1.12–5.01) compared with good PWB.

**Conclusion:**

In women with advanced breast cancer, PWB and appetite are predictors of chemotherapy response and toxicity as well as survival. Quality of life should be a routine clinical assessment to guide patient selection for chemotherapy and for stratification of patients in clinical trials.

In advanced breast cancer, the primary goals of treatment are to prolong and improve quality of life (QL) because the disease is generally incurable. In a pivotal study conducted by Coates *et al* (1987), patient-rated QL improved significantly on average in women with advanced breast cancer receiving palliative chemotherapy, and both baseline QL scores and subsequent changes were prognostic for survival (Coates *et al*, 1992). Although chemotherapy is effective in relieving cancer-related symptoms, these average benefits may not accrue to all patients and may in some cases be offset by the significant physical and psychological side-effects. Therefore, appropriate selection of patients for chemotherapy is important to increase the likelihood that its benefits will likely outweigh its harms.

Determination of the prognosis of patients with advanced breast cancer can provide valuable information to guide oncologists in deciding whether to offer palliative chemotherapy. Oncologists consider a range of patient and disease characteristics such as age, performance status, disease-free interval, hormone receptor status, disease burden and prior adjuvant treatments (Hortobagyi *et al*, 1983; Gennari *et al*, 2005; Largillier *et al*, 2008). More recently, patient's self-reported health-related QL has also been recognised as predictive of survival (Gotay *et al*, 2008; Quinten *et al*, 2009).

Poor QL before treatment is associated with shorter survival in patients with advanced breast cancer (Coates *et al*, 1992; Efficace *et al*, 2004; Winer *et al*, 2004; Gotay *et al*, 2008). However, there is only limited evidence of the relationship between baseline QL and response to chemotherapy (Seidman *et al*, 1995; Kramer *et al*, 2000; Winer *et al*, 2004) and treatment-related toxicity. Investigation of this issue is important because chemotherapy has a narrow therapeutic index and a fine balance between benefits and harms. Such information might help oncologists and patients to individualise their treatment decisions.

In this study, we used data from three trials conducted by the Australian New Zealand Breast Cancer Trials Group (ANZBCTG) to validate earlier findings that baseline QL predicts overall survival (OS) in advanced breast cancer and investigate the association between baseline QL and chemotherapy response and toxicity.

## Materials And Methods

We used data from the common control arms of three randomised controlled trials (RCTs) of first-line chemotherapy for patients with advanced breast cancer conducted by ANZBCTG (ANZ8101, ANZ8614 and ANZ0001). Trial participants were recruited from participating hospitals across Australia and New Zealand. ANZ8101, activated in June 1982, was a two-by-two factorial RCT comparing the efficacy of doxorubicin and cyclophosphamide *vs* cyclophosphamide, methotrexate, 5-fluouracil and prednisone (CMFP), administered continuously *vs* intermittently (Coates *et al*, 1987). ANZ8614, activated in January 1988, was a two-arm RCT comparing the efficacy of mitoxantrone *vs* CMFP (Simes *et al*, 1994). ANZ0001, activated in June 2001, was a three-arm RCT comparing the efficacy of intermittent capecitabine *vs* continuous capecitabine *vs* CMFP (Stockler *et al*, 2007).

All three trials included a measurement of patients’ self-reported QL at baseline. Investigation of QL as a predictor of treatment response or toxicity was not specified in the original trial protocols.

### Patients

Patients had histologically confirmed breast carcinoma with measurable or evaluable recurrent or metastatic disease; adequate bone marrow, hepatic and renal function; and were available for follow-up. Patients were excluded if they had received cytotoxic chemotherapy for recurrent or metastatic disease or extensive radiotherapy, or had a history of other cancer, diabetes mellitus or cardiac failure.

Only patients assigned to the continuous CMFP in each of the three trials were included in the present analysis. Patients assigned to the intermittent CMFP arm of ANZ8101 were excluded from this study because this treatment arm was inferior to the CMFP regimen given continuously as in the other trials. All patients provided written informed consent for participation in the trials.

### Treatments

In each of the three trials, CMFP was administered in 28-day cycles with oral cyclophosphamide (100 mg m^−2^) daily for 14 days; intravenous methotrexate (40 mg m^−2^) and intravenous 5-fluorouracil (600 mg m^−2^) on days 1 and 8. Oral prednisone (40 mg m^−2^) for first 14 days was routinely administered in patients from ANZ8101 and ANZ8614, and was optional in ANZ0001. All patients continued the initial chemotherapy regimen until disease progression, intolerance or unacceptable toxicity. Therapy beyond disease progression was at the discretion of the treating oncologist.

### QL instruments

Patients self-reported their QL with five linear analogue self assessment (LASA) scales that measured physical well-being (PWB), mood, pain, nausea/vomiting and appetite (Priestman *et al*, 1977; Coates *et al*, 1993), and a single summative LASA scale that measured overall QL (Spitzer *et al*, 1981). All scales were 100 mm long and scores range from 0 (best) to 100 (worst).

For the purpose of our analysis, scores for each QL scale were arbitrarily divided into three groups: good (0–25), mid (26–65) or poor (66–100). The categorisation was selected to be consistent with the cut-points used in a previous study (Coates *et al*, 1992).

### Treatment benefits

Treatment benefits were evaluated by measuring OS, progression-free survival (PFS), objective tumour response (OTR) and improvement in baseline body weight. OS and PFS were measured from randomisation to the date of death or first documented disease progression respectively. OTR rate was measured as the proportion of patients with evaluable disease who achieved a complete response (CR) or partial response (PR). Weight loss (*vs* stable or weight gain) was measured as the proportion of patients with an average decrease in weight from their baseline reading.

### Treatment toxicity

Adverse events (AEs) were expressed as the proportion of patients who developed any grade 3 or grade 4 toxicity during the first 4 cycles of chemotherapy treatment. World Health Organisation criteria (Miller *et al*, 1981) were used for ANZ8101 and ANZ8614; National Cancer Institute Common Toxicity Criteria (Arbuck *et al*, 1998) were used for ANZ0001. Haematological (anaemia, neutropenia and thrombocytopenia) and non-haematological (nausea, vomiting, diarrhoea, stomatitis and alopecia) AEs were analysed separately.

### Statistical analysis

Overall survival and PFS were estimated by the Kaplan–Meier method, and differences between patients with good, mid and poor levels for each QL scale at baseline were compared with the log-rank test (Kaplan and Meier, 1958). Cox proportional-hazard models were used to estimate differences in OS according to each level of the QL scale (Cox, 1972). Multivariable analysis for OS was first performed with backward stepwise selection of biomedical variables only. Then, multivariable analysis for OS was repeated with backward stepwise selection of biomedical and QL variables. Only statistically significant variables (*P*<0.05) were retained in the final multivariable models. As shown in a previous analysis using the same data set (Lee *et al*, 2010), trial is a significant factor for survival; thus we stratified for trial in all univariable and multivariable analyses for this study. Formal tests to detect the presence of collinearity between the different QL scales, and QL scales and ECOG performance status were also performed (Weissfeld, 1989).

Univariable analyses with logistic regression tested for associations between QL subscales and OTR, AE and weight loss. Multivariable models for these outcomes were constructed to estimate the effects of these QL subscales, adjusted for biomedical variables found to be significant in Cox model for OS.

All analyses were two-sided with no adjustment for multiple comparisons. No imputation of missing baseline QL values was performed.

## Results

### Biomedical and demographic characteristics

A total of 378 patients with a median follow-up of 4.8 years were included in this pooled analysis. The number of patients from ANZ8101, ANZ8614 and ANZ0001 were 75 (20%), 194 (51%) and 109 (29%), respectively. The median follow-up of these patients from ANZ8101, ANZ8614 and ANZ0001 were 4.8 years (range 0–4.8 years), 10.1 years (range 0–10.9 years) and 3.2 years (range 0–4.8 years), respectively. Table 1 summarises the baseline characteristics and the treatment profiles of these patients for the overall cohort and according to the baseline PWB. Apart from performance status and lung metastasis, distribution of baseline characteristics was similar in patients with good, mid and poor PWB. Similar patterns of baseline characteristics were observed for other QL scales (data not shown).

### Baseline QL scores

Baseline LASA scores for each QL scale were available for 89–94% of the patients. The proportion of patients with self-rating for each scale was 93% (PWB), 94% (mood), 93% (pain), 93% (nausea and vomiting), 93% (appetite) and 89% (overall life quality). The distribution of the three categories of LASA scores for each QL subscale is shown in Table 2B.

### CMFP treatment

Two patients randomised to CMFP chemotherapy in the original trials did not receive the assigned treatment. The remaining 376 patients (99.5%) received a median five cycles of treatment. The chemotherapy doses administered were between 90 and 93% of those planned. Patients with good, mid and poor PWB received medians of 6, 5 and 3 cycles of CMFP, respectively (Table 1).

### OS and PFS

**Figure 1:**
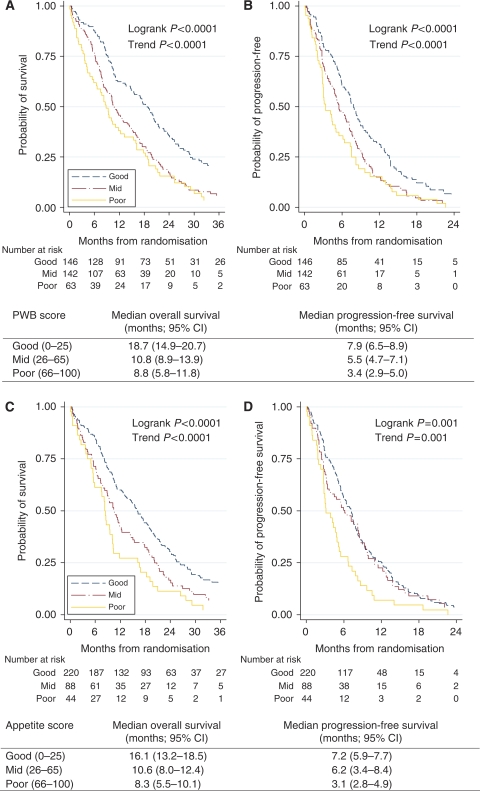
Overall survival (**A**) and PFS (**B**) curves stratified by PWB score, and OS (**C**) and PFS (**D**) curves stratified by Appetite score.

Physical well-being, mood, appetite and overall QL were predictors of OS in the univariable analyses (Table 2B). Physical well-being and appetite were independent predictors of survival in a multivariable model with biomedical and QL variables (Table 3B). Performance status was statistically significant in a multivariate model with biomedical factors only (Table 3A), but was not statistically significant in a multivariable model with biomedical and QL scales (Table 3B). Patients with good PWB at baseline had a statistically significantly longer PFS and OS compared with patients who had mid or poor PWB (Figure 1A and B). The median OS for patients with good, mid and poor PWB were 19, 11 and 9 months, respectively (log-rank *P*<0.0001). Similar results were observed for patients with good, mid and poor appetite (Figure 1C and D).

Significant collinearity was not detected between the different QL scales or between QL scales and ECOG performance status (results not shown).

### Objective tumour response

**Figure 2:**
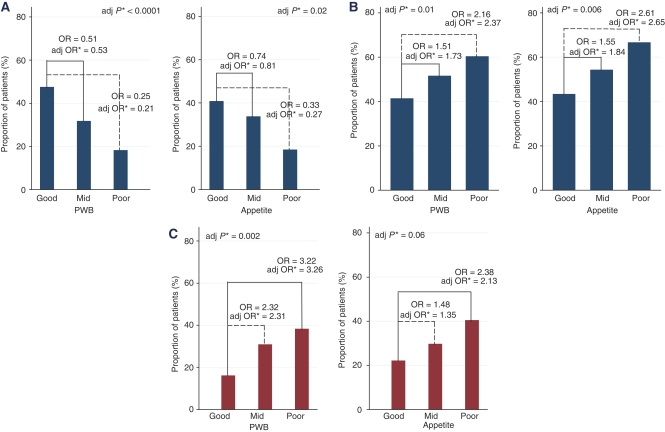
(**A**) Proportion of OTR stratified by PWB and Appetite scores. (**B**) Proportion with weight loss during chemotherapy stratified by PWB and Appetite scores. (**C**) Proportion with grade-3/4 non-haematological toxicity stratified by PWB and Appetite scores (^*^QL adjusted for performance status, age, liver, and brain metastasis, oestrogen receptor status, neutrophil, serum alkaline phosphatase and trial enrolment).

The OTR rates for patients with good, mid and poor PWB were 47, 32 and 18%, respectively (adjusted *P*_trend_<0.001; Figure 2A). For patients with good, mid and poor appetite, the OTR rates were 41, 34 and 18% respectively (adjusted *P*_trend_=0.02; Figure 2A).

### Weight loss

Fifty-one percent of women experienced weight loss during chemotherapy. Women with good PWB had a mean weight gain during chemotherapy of 2%, but mean weight loss was 2% for women with mid PWB and 4% for women with poor PWB, respectively (adjusted *P*_trend_=0.01; Figure 2B). For women with good appetite, there was a mean weight gain of 0.5%, but mean weight loss was 2% for women with mid appetite and 7% for women with poor appetite (adjusted *P*_trend_=0.006; Figure 2B).

### Treatment toxicity

Non-haematological AE rates were statistically significantly different for patients with good (16%), mid (31%) and poor (38%) PWB (adjusted *P*_trend_=0.002; Figure 2C). Grade-3 or 4 nausea and vomiting were reported in 19, 41 and 42% diarrhoea in 9, 12 and 11% and grade-3 and 4 stomatitis in 8, 11 and 17% of patients with good, mid and poor PWB, respectively. Grade-3 and 4 alopecia rates were similar in the three PWB groups.

The rates of non-haematological AEs were not significantly different in patients with good, mid and poor appetite (adjusted *P*_trend_=0.06; Figure 2B).

Grade-3 and 4 haematological toxicity rates were not associated with PWB (adjusted *P*_trend_=0.92) or appetite scores (adjusted *P*_trend_=0.60).

## Discussion

In this analysis of women treated with CMFP as first-line chemotherapy for advanced breast cancer, patient self-reported PWB and appetite at baseline were independent predictors of OS and PFS. Women with poor PWB had a median OS that was 10 months shorter, and a median PFS that was 5 months shorter, than women with good PWB. Furthermore, women with poor PWB had OTR rates 29% lower, weight loss rates 19% lower and treatment-related non-haematological AE rates 22% higher than women with good PWB. Similar findings were observed for women with poor appetite compared with those who had good appetite.

Our study also showed that women with poor QL derived less benefit and experienced more toxicity when treated with chemotherapy than women with good QL. Overall, women reporting poor QL at baseline received 50% fewer cycles of chemotherapy, were 20–30% less likely to benefit from chemotherapy and experienced rates of non-haematological treatment toxicity 20% higher than women reporting good QL at baseline.

This research adds to the growing evidence that QL is an independent predictor of survival in advanced cancer. In a systematic review of cancer trials, Gotay *et al* (2008) reported that 36 of 39 trials showed an association between QL and survival. Specific QL measures most frequently identified in these trials were overall QL, physical well being, appetite loss and pain. Another meta-analysis using data from 30 cancer trials produced a multivariable model that identified impaired physical functioning, pain and appetite loss as independent predictors of survival in addition to established biomedical factors (Quinten *et al*, 2009). Individual trials in advanced breast cancer have shown similar findings with PWB (Coates *et al*, 1992), appetite loss (Efficace *et al*, 2004), pain (Kramer *et al*, 2000) and overall QL (Seidman *et al*, 1995; Winer *et al*, 2004) all reported as independent predictors of survival. This study validates the prognostic significance of self-reported QL, and specifically of PWB and appetite, as predictors of survival in advanced breast cancer.

Furthermore, our findings raise the important question of whether measurement of baseline QL can be used to improve the selection of patients for chemotherapy. To date, few studies have investigated baseline QL as predictor of treatment benefit and/or toxicity in advanced breast cancer. Kramer *et al* (2000) reported an association between QL (dyspnoea, fatigue and overall QL) and tumour response in their analysis of 187 women treated with paclitaxel or doxorubicin, but selection of specific QL scales as independent predictors in the final multivariable model was reported as unstable because of multi-collinearity. They did not examine associations between QL and toxicity in this analysis. Two other studies reported no association between QL and tumour response to chemotherapy (Seidman *et al*, 1995; Winer *et al*, 2004) or treatment-related toxicity (Seidman *et al*, 1995). Possible explanations for the differences between these results and our study include small sample size (Seidman *et al*, 1995), the type of QL instruments and QL subscales used, and the methods used to assess tumour response.

The main strength of this study is the use of high-quality, individual patient data from three successive randomised clinical trials conducted by the same group. The pooled data set contains well-documented demographic, clinical and QL characteristics of trial participants who were treated with the same regimen of chemotherapy. Women in this study were assigned chemotherapy independent of their baseline QL. Follow-up and outcome data were collected prospectively with rigorous quality control.

This study has several limitations. First, our findings are from women with advanced breast cancer who were treated with CMFP. They may differ with other chemotherapy regimens or for other cancers (Quinten *et al*, 2009). Second, the results are not generalisable to women with early-stage breast cancer who do not have tumour-related symptoms (Coates *et al*, 2000; Goodwin *et al*, 2004; Gotay *et al*, 2008). Finally, this analysis was conducted *post hoc* using available trial data and therefore should be regarded as hypothesis-generating for future studies, rather than definitive.

If our findings are confirmed, trials of new treatment approaches should be investigated for women with poor QL. These trials could address the value of any chemotherapy *vs* none, of less intense *vs* standard-intensity chemotherapy, or of chemotherapeutic or biological agents with a more favourable therapeutic–toxic ratio. Outcomes, which incorporate both survival time and QL, such as quality-adjusted PFS and OS, may be more relevant measures of treatment benefit in these women. The results of this study also suggest the value of stratifying patients by their baseline QL for future randomised trials in advanced breast cancer.

The primary goal of chemotherapy in advanced breast cancer is to prolong and improve QL. Our findings suggest that women with poor QL derive less benefit from chemotherapy and have increased risks of toxicity than women with good QL. This analysis should be regarded as hypothesis-generating and future studies that examine the role of chemotherapy in advanced breast cancer patients with poor QL are warranted.

## Figures and Tables

**Table 1:** Demographic characteristics and chemotherapy treatment profile

**Characteristics**	**Overall *n* (%)**	**Good PWB^a^ *n* (%)**	**Mid PWB^a^ *n* (%)**	**Poor PWB^a^ *n* (%)**
Age>60 years	170 (45)	69 (47)	63 (44)	26 (41)
Post-menopausal	275 (73)	108 (74)	105 (74)	44 (70)
				
*Performance status*				
0	123 (32)	69 (47)	37 (26)	11 (18)
1	153 (40)	54 (37)	69 (49)	21 (33)
2	74 (20)	16 (11)	30 (21)	21 (33)
3	25 (7)	7 (5)	6 (4)	8 (13)
4	2 (1)	0 (0)	0 (0)	2 (3)
				
*Extent of advanced cancer*				
Local or regional disease only	46 (12)	20 (14)	16 (11)	6 (9)
Distant disease only	186 (49)	73 (50)	70 (49)	32 (51)
Locoregional and distant disease	146 (39)	53 (36)	56 (40)	25 (40)
Disease-free interval >2years	208 (55)	78 (53)	74 (52)	38 (60)
				
*Hormone receptor status of primary breast tumour*				
ER+	151 (40)	63 (43)	56 (39)	25 (40)
ER−	101 (27)	38 (26)	37 (26)	20 (32)
PR+	115 (30)	47 (32)	43 (30)	18 (29)
PR−	111 (29)	43 (29)	42 (30)	22 (35)
				
*Tissue sites of metastasis* b				
Liver	148 (39)	56 (38)	50 (35)	26 (41)
Lung	123 (33)	32 (22)	59 (42)	25 (40)
Brain	12 (3)	4 (3)	4 (3)	3 (5)
Bone	256 (68)	96 (66)	99 (70)	44 (70)
Prior adjuvant chemotherapy	84 (22)	29 (20)	31 (22)	20 (32)
Prior endocrine therapy	292 (77)	108 (74)	108 (76)	54 (86)
Haemoglobin⩽12 g dl^−1^	132 (35)	42 (29)	52 (37)	24 (38)
Neutrophil>7.5 × 10^9^ l^−1^	46 (13)	14 (10)	16 (12)	9 (15)
Bilirubin>15 *μ*mol l^−1^	26 (7)	13 (9)	4 (3)	6 (10)
Alkaline phosphatase>125 IU l^−1^	199 (54)	66 (46)	79 (57)	38 (62)
Median number of cycles of CMFP treatment	5	6	5	3
Range of cycles of CMFP treatment	1–25	1–25	1–21	1–14
Percentage of cyclophosphamide received^c^	90%	92%	88%	83%
Percentage of methotrexate received^c^	92%	96%	89%	81%
Percentage of 5-fluouracil received^c^	93%	95%	92%	81%

Abbreviations: CMFP=cyclophosphamide, methotrexate, 5-fluouracil, prednisone chemotherapy; ER=oestrogen receptor; PR=progesterone receptor; PWB=physical well-being.

a Good PWB=LASA score 0–25; mid PWB=LASA score 26–65; poor PWB=LASA score 66–100.

b More than one site could have been involved, so percentages sum to more than 100%.

c The reported percentage is the median total dose of chemotherapy received as compared with the ideal dose calculated on the basis of the body-surface area.

**Table 2:** Univariable Cox regression analysis of survival (*n*=378)

	***n* (%)**	**Hazard ratio**	**(95% CI)**	***P*-value**
*(A) Baseline demographic and clinical characteristics*				
Age >60 (*vs* ⩽60 years)	170 (45)	1.29	(1.04–1.60)	0.02
Post-menopausal	275 (73)	1.21	(0.95–1.55)	0.12
				
*Performance status (PS)*				
PS 0	123 (32)	1.00	—	0.0007
PS 1	153 (40)	1.40	(1.09–1.80)	
PS2	74 (20)	1.65	(1.21–2.23)	
PS3 & PS4	27 (8)	2.08	(1.35–3.21)	
Disease-free interval > 2 years	208 (55)	1.19	(0.95–1.48)	0.13
				
*Prior treatment*				
Prior adjuvant chemotherapy	84 (22)	0.89	(0.68–1.17)	0.4
Prior endocrine therapy	292 (77)	1.20	(0.94–1.55)	0.15
				
*Hormone receptor status*				
ER−	101 (27)	1.00	—	0.01
ER+	151 (40)	0.72	(0.54–0.94)	
ER unknown	126 (33)	1.00	(0.77–1.32)	
PR−	111 (29)	1.00	—	0.01
PR+	115 (30)	0.85	(0.65–1.13)	
PR unknown	152 (40)	1.27	(0.98–1.65)	
				
*Sites of metastasis*				
Presence of liver metastasis (*vs* none)	148 (39)	1.33	(1.07–1.66)	0.01
Presence of brain metastasis (*vs* none)	12 (3)	2.53	(1.37–4.67)	0.003
Presence of lung metastasis (*vs* none)	123 (33)	1.40	(1.11–1.75)	0.004
Presence of bone metastasis (*vs* none)	256 (68)	0.94	(0.75–1.17)	0.57
Haemoglobin⩽12g dl^−1^	132 (35)	1.23	(0.99–1.53)	0.07
Neutrophil>7.5 × 10^9^ l^−1^	46 (13)	1.88	(1.36–2.60)	<0.001
Bilirubin>15 *μ*mol l^−1^	26 (7)	1.89	(1.24–2.87)	0.003
Alkaline phosphatase >125 IU l^−1^	199 (54)	1.31	(1.05–1.63)	0.02
				
*(B) Baseline QL*^a^ *(LASA variables)*				
*Physical well-being (PWB)*				<0.0001
Mid PWB (*vs* good PWB)	142 (40)	1.70	(1.33–2.18)	
Poor PWB (*vs* good PWB)	63 (18)	2.04	(1.50–2.78)	
				
*Appetite*				<0.0001
Mid appetite (*vs* good appetite)	88 (25)	1.60	(1.23–2.08)	
Poor appetite (*vs* good appetite)	44 (13)	2.10	(1.50–2.93)	
				
*Overall QL*				0.0001
Mid overall (*vs* good overall)	142 (42)	1.71	(1.34–2.19)	
Poor overall (*vs* good overall)	37 (11)	1.41	(0.96–2.06)	
				
*Mood*				0.01
Mid mood (*vs* good mood)	151 (43)	1.44	(1.13–1.83)	
Poor mood (*vs* good mood)	48 (14)	1.31	(0.94–1.84)	
				
*Pain*				0.08
Mid pain (*vs* good pain)	113 (32)	1.16	(0.90–1.49)	
Poor pain (*vs* good pain)	63 (18)	1.41	(1.05–1.91)	
				
*Nausea/vomiting*				0.21
Mid nausea/vomiting (*vs* good nausea/vomiting)	49 (14)	1.30	(0.95–1.77)	
Poor nausea/vomiting (*vs* good nausea/vomiting)	14 (4)	1.29	(0.73–2.28)	

Abbreviations: CI=confidence interval; ER=oestrogen receptor; LASA=linear analogue self-assessment; PR=progesterone receptor; QL=quality of life.

a Good QL=LASA score 0–25, Mid QL=LASA score 26–65, Poor QL=LASA score 66–100.

**Table 3:** Multivariable Cox regression analyses of survival for demographic and baseline clinical characteristics, and for baseline demographic and clinical characteristics and QL variables (*n*=378)

	**Hazard ratio**	**(95% CI)**	***P*-value**
*(A) ‘Best’ model with demographic and clinical characteristics only*			
Age >60	1.46	(1.16–1.84)	0.001
ER−	1.00	—	0.002
ER+	0.63	(0.47–0.84)	
ER unknown	0.94	(0.70–1.25)	
Neutrophil>7.5 × 10^9^ l^−1^	1.68	(1.18–2.40)	0.004
PS 0	1.00	—	0.005
PS1	1.44	(1.11–1.88)	
PS2	1.60	(1.16–2.22)	
PS3 & PS4	1.83	(1.14–2.93)	
Presence of brain metastasis (*vs* none)	2.42	(1.28–4.57)	0.006
Presence of liver metastasis (*vs* none)	1.38	(1.08–1.77)	0.009
Alkaline phosphatase > 125 IU l^−1^	1.31	(1.03–1.67)	0.03
			
*(B) ‘Best’ model with demographic, clinical characteristics and QL variables*			
Neutrophil>7.5 × 10^9^ l^−1^	1.91	(1.31–2.79)	0.001
Age>60	1.45	(1.14–1.83)	0.002
ER−	1.00	—	0.004
ER+	0.64	(0.47–0.87)	
ER unknown	0.98	(0.72–1.33)	
Good PWB^a^	1.00	—	0.004
Mid PWB^a^	1.45	(1.09–1.91)	
Poor PWB^a^	1.64	(1.12–2.40)	
Presence of brain metastasis (*vs* none)	2.37	(1.21–4.64)	0.01
Alkaline phosphatase >125 IU l^−1^	1.37	(1.08–1.74)	0.01
Good appetite^a^	1.00	—	0.03
Mid appetite^a^	1.36	(1.01–1.84)	
Poor appetite^a^	1.49	(0.98–2.24)	

Abbreviations: CI=confidence interval; ER=oestrogen receptor; PWB=physical well-being; PS=performance status; QL=quality of life; ULN=upper limit normal.

a Good PWB/appetite=LASA score 0–25; mid PWB/appetite=LASA score 26–65; poor PWB/appetite=LASA score 66–100.
